# Supplementary material for: Utility of the monocyte to lymphocyte ratio in diagnosing latent tuberculosis among HIV-infected individuals with a negative tuberculosis symptom screen
Source: PLoS One. 2020 Nov 9;15(11):e0241786. doi: 10.1371/journal.pone.0241786 (PMC7652277; doi:10.1371/journal.pone.0241786)
Supplement: S1 File — (PDF) [file pone.0241786.s001.pdf]

MAKERERE UNIVERSITY
P.O. Box 7072 Kampala, Uganda
E-mail: biomedicalresearch62@gmail.com
Phone:256752575050
Fax: 256 414 532204

COLLEGE OF HEALTH SCIENCES
SCHOOL OF BIOMEDICAL SCIENCES
RESEARCH AND ETHICS COMMITTEE

20th July 2020

SBS-794

To Mr. Mayito Jonathan
Principal Investigator
Infectious Diseases Institute

Category of review
[X] Initial review
[ ] Continuing review
[ ] Amendment
[ ] Termination of study
[ ] SAEs

Decision of the School of Biomedical Sciences Research and Ethics Committee (SBS-REC) at its 100th REC meeting held on 25th June 2020.

In the matter concerning the review of a proposal entitled, **"Utility of the monocyte ration in diagnosing latent TB and HIV- infected individuals with a negative TB symptom screen." SBS-REC - 794**

The investigators have met all the requirements as stated by **SBS-REC** and therefore, the proposal is **APPROVED.**

The approval granted includes all materials submitted by the investigators for **SBS-REC** review including;

1. Protocol version 1.4 of 15/07/2020
2. English participation informed consent form of 15/07/2020
3. Luganda participation informed consent form of 15/07/2020
4. Case Report forms (phase 1 and 2) version 1.3 of 3/05/2020

and is valid until **24th June 2021**

Please note that the annual report and the request for renewal where applicable, should be submitted six weeks before expiry date of approval.

Any problems of a serious nature related to the execution of the research protocol should be promptly reported to the **SBS-REC**, and any changes to the research protocol should not be implemented without approval from **SBS-REC**, except when necessary to eliminate apparent immediate hazards to the research participant(s)

You are required to register the research protocol with the Uganda National Council for Science and Technology (**UNCST**) for final clearance to undertake the study in Uganda.

Signed...

MAKERERE UNIVERSITY
SCHOOL OF BIOMEDICAL SCIENCES
APPROVED
VALID UNTIL
24 JUN 2021
RESEARCH & ETHICS COMMITTEE
P. O. BOX 7072, KAMPALA

Dr. Jackson Mukonzo
Vice Chairperson, School of Biomedical Sciences Research and Ethics Committee.
